# Acyl-CoA Synthetase Long-Chain Family Member 4 in Liver Injury: Multidimensional Regulation and Therapeutic Potential

**DOI:** 10.14740/gr2098

**Published:** 2026-01-04

**Authors:** Ming Xing Liang, Mao Yi Wang, Yu Zhi Su, Ying Zhou, Yu Xin Xie, Wei Li, Ying Hua Chen, Yi Huai He

**Affiliations:** a Department of Infectious Diseases, Affiliated Hospital of Zunyi Medical University, Zunyi 563000, Guizhou Province, China; b Department of Infectious Diseases, Wenjiang District People’s Hospital, Chengdu 611130, Sichuan Province, China; c These authors contributed equally to this work.

**Keywords:** Acyl-CoA synthetase long-chain family member 4, Liver injury, Ferroptosis, Lipid peroxidation, Therapeutic potential

## Abstract

Acyl-CoA synthetase long-chain family member 4 (ACSL4) is a key enzyme that catalyzes the conjugation of long-chain fatty acids with coenzyme A to form acyl-CoA, showing particularly high specificity for polyunsaturated fatty acids. In recent years, ACSL4 has gained increasing attention for its central role in various liver diseases, including metabolic dysfunction-associated steatotic liver disease, liver fibrosis, hepatocellular carcinoma, and ferroptosis. This article systematically elaborates on the expression profiles and localization of ACSL4 in different liver cell types, as well as its multidimensional regulatory mechanisms in liver injury and the pathogenesis of related diseases. In addition, it explores the potential therapeutic prospects of targeting ACSL4.

## Introduction

Liver diseases account for approximately 2 million deaths annually, representing 4% of global mortality. The most common etiologies include metabolic dysfunction-associated steatotic liver disease (MASLD), alcoholic liver disease (ALD), drug-induced liver injury (DILI), and viral hepatitis [1, 2]. Recent systematic reviews indicate that the global average prevalence of MASLD has risen to 30% and continues to increase, with a parallel acceleration in the progression to metabolic dysfunction-associated steatohepatitis (MASH) [3, 4]. DILI is also a significant cause of acute liver injury, liver failure, and transplantation; its incidence is on the rise, particularly in the context of novel therapies, such as immune checkpoint inhibitors [5]. Viral hepatitis, especially hepatitis B virus (HBV) and hepatitis C virus (HCV), contributes to about 1.3 million deaths annually [2]. In addition, liver ischemia-reperfusion injury (LIRI) remains an unavoidable and critical complication in transplantation and hepatic resection surgeries, significantly impairing perioperative outcomes [6]. Although these liver injuries vary in pathophysiology, clinical manifestations, and therapeutic strategies, all can progress to fibrosis, cirrhosis, and even hepatocellular carcinoma (HCC), posing severe threats to patient health and imposing substantial socioeconomic burdens. Currently, effective treatments for many forms of liver injury remain limited, partly due to incomplete understanding of their complex pathogenic mechanisms. This gap represents a major clinical challenge.

Hepatocytes and non-parenchymal cells show diverse death modalities, primarily including apoptosis, necrosis, and various regulated cell death pathways, such as necroptosis, pyroptosis, and ferroptosis [7, 8]. Recent studies have identified acyl-CoA synthetase long-chain family member 4 (ACSL4) as a key positive regulator of ferroptosis [9, 10]. ACSL4 facilitates ferroptosis by catalyzing the activation of polyunsaturated fatty acids (PUFAs), such as arachidonic acid (AA), and cooperating with lysophosphatidylcholine acyltransferase 3 to esterify them into oxidizable phosphatidylethanolamine (PUFA-PE), thereby providing lipid substrates for ferroptosis. Under conditions of glutathione peroxidase 4 (GPX4) dysfunction and iron overload, these lipids undergo extensive peroxidation, disrupting membrane integrity and driving cell death [11]. Thus, ACSL4 determines cellular susceptibility to ferroptosis by modulating lipid metabolism and plays a critical role in various liver diseases, such as ALD, MASH, and HCC [12-14].

Furthermore, as a key regulator of lipid metabolism, ACSL4 functions as a “gatekeeper” [15, 16]. Its core physiological role involves activating long-chain free fatty acids into fatty acyl-CoA, thus providing essential substrates for cellular energy metabolism, membrane lipid synthesis, and signal transduction. By participating in critical processes such as mitochondrial β-oxidation, phospholipid remodeling, and steroid synthesis, ACSL4 plays a central role in maintaining hepatic energy homeostasis and membrane structural integrity. This extensive regulation of lipid metabolic pathways positions ACSL4 as a crucial factor in preserving hepatic lipid homeostasis and defending against lipotoxic injury.

Against this background, this review aims to systematically summarize current research advances regarding the specific mechanisms of ACSL4 in liver injury induced by various etiologies. We will provide an in-depth discussion of its role as both a driver of liver injury progression and a potential therapeutic target with clinical significance. By integrating both classical and cutting-edge high-quality research articles and reviews in the field, we seek to provide a comprehensive perspective for further elucidating the complex roles of ACSL4 in liver pathophysiology and offer theoretical foundations for developing novel ACSL4-targeted therapeutic strategies against liver injury.

## *ACSL4* Gene, Protein Structure, and Enzymatic Characteristics

### Genomic architecture and splice isoforms of *ACSL4*

The *ACSL4* gene is located on the human X chromosome at position Xq22.3-q23, spanning about 90 kb with multiple exons and introns [17]. Early cDNA cloning and fluorescence *in-situ* hybridization mapping studies revealed that this gene generates two major transcript variants through alternative splicing. Variant 1 encodes a 670-amino acid protein, and variant 2 contains an additional 41-amino acid insertion at the N-terminus, resulting in a 711-amino acid protein [18, 19]. Notably, variant 2 shows nervous system-specific expression and uses its novel hydrophobic N-terminal segment to mediate targeting to the endoplasmic reticulum and lipid droplets [18].

### Protein domain architecture and subcellular localization signals

The ACSL4 polypeptide comprises about 670 residues (about 74.4 kDa) and can be structurally divided into five functional domains, namely an NH_2_-terminal region, two luciferase-like regions (LR1 and LR2), a linker domain, and a COOH-terminal region, with the highly conserved LR2 and COOH-terminal sequences forming the catalytic core responsible for substrate cross-linking [20]. ACSL4 is widely distributed across multiple subcellular structures, primarily localized in the endoplasmic reticulum, outer mitochondrial membrane, peroxisomal membrane, and microsomal membrane [20-22]. Notably, it is specifically enriched on the mitochondria-associated endoplasmic reticulum membrane (MAM), consistent with the central role of this compartment in lipid metabolism. ACSL4 is reportedly mainly localized in MAM and significantly contributes to acyl-CoA synthetase activity in this region; for instance, N-ethylmaleimide and troglitazone inhibit acyl-CoA synthetase activity in the MAM by 47% and 45%, respectively [21, 23]. In addition, ACSL4 is distributed in the endoplasmic reticulum and peroxisomes, where it participates in lipid synthesis and is essential for normal steroid hormone biosynthesis [24], while also contributing to the maintenance of cell membrane fluidity [25]. Beyond these compartments, ACSL4 has been detected on the plasma membrane and in endosomes [20]; in addition to its role in lipid synthesis, it can promote endometrial placental formation by activating the β-oxidation pathway [26]. Post-translational modifications (PTMs), such as phosphorylation, may regulate the subcellular distribution of ACSL4, thus influencing its intracellular targeting and catalytic activity [17]. Functionally, ACSL4 restores fatty acid transport activity and promotes triglyceride synthesis, and its expression is regulated by nutritional status [23, 27]. Furthermore, variant 2 of ASCL4 can target the endoplasmic reticulum and the surface of intracellular lipid droplets via an additional N-terminal hydrophobic segment, suggesting functional diversity in its participation across lipid metabolic pathways under different cell types and physiological states [28].

### Enzymatic activity, substrate preference, and metabolic regulation

ACSL4, as the key rate-limiting enzyme in long-chain fatty acid metabolism, catalyzes the conjugation of fatty acids with CoA to form acyl-CoA, thus determining the metabolic fate of fatty acids in downstream pathways [29]. Its most remarkable enzymatic characteristic is the pronounced substrate preference for PUFAs, particularly showing high selectivity toward AA, eicosapentaenoic acid (EPA, 20:5n-3), and adrenic acid (AdA) [30, 31]. Notably, the catalytic efficiency of ACSL4 for AA is significantly higher than that for other PUFAs (e.g., linoleic acid) [32]. In addition, distinct Vmax variations are observed among different PUFA substrates [28], suggesting potential substrate “channeling” preferences and tissue-specific functional specialization.

This unique substrate specificity establishes ACSL4 as a central regulator in PUFA metabolic networks. Mechanistically, ACSL4 preferentially activates AA and other PUFAs into their CoA derivatives (e.g., AA-CoA), directly modulating inflammatory responses, cellular signaling, and ferroptosis [33]. Intriguingly, AA, as the native substrate, induces ubiquitin-mediated degradation of ACSL4, reducing its half-life from about17.3 h to 4.2 h in HepG2 cells through enhanced proteasomal degradation (a substrate-driven feedback mechanism for enzymatic homeostasis) [34]. During ferroptosis, ACSL4-generated PUFA-CoA products are incorporated into phospholipids, serving as substrates for lipid peroxidation cascades that amplify membrane damage and drive cell death [35, 36]. Pathologically, hepatic ACSL4 is markedly upregulated in MASLD, with expression levels correlating positively with disease severity. ACSL4 inhibition restores mitochondrial respiration and β-oxidation capacity, thus ameliorating hepatic steatosis [37]. These findings collectively unveil the dual regulatory mechanisms governing the enzymatic activity and protein stability of ACSL4 across physiological and pathological contexts, providing a robust rationale for developing ACSL4-targeted therapies against MASLD.

## Regulatory Networks of ACSL4

Recent studies have shown that ACSL4 expression and function are regulated at multiple levels: 1) transcriptionally by nuclear receptors including peroxisome proliferator-activated receptor delta (PPARδ) and sterol regulatory element-binding protein 2 (SREBP2) [38, 39]; 2) post-transcriptionally through miRNA, circRNA, and N6-methyladenosine (m^6^A) modifications that modulate mRNA stability and translation efficiency [40-42], post-translationally via ubiquitination and phosphorylation modifications that regulate protein stability and enzymatic activity [43, 44]. Meanwhile, substrates like AA and metabolites like cyclic adenosine monophosphate (cAMP) can feedback-regulate ACSL4 degradation and expression levels [34, 45], collectively forming a multidimensional regulatory network. These integrated mechanisms establish the pivotal role of ACSL4 in mediating lipid metabolic reprogramming and determining cellular fate during liver injury and other pathological processes.

### Transcriptional regulation

#### PPARδ

Studies have shown that PPARδ agonists significantly enhance ACSL4 transcriptional activity by directly activating its promoter, resulting in increased expression of ACSL4 mRNA and protein in hepatic tissues and HepG2 cells, along with markedly elevated AA-CoA synthetase activity [39]. Mechanistically, PPARδ-mediated regulation of ACSL4 acts synergistically with the upregulation of lysophosphatidylcholine acyltransferase 3, forming a coordinated pathway that contributes to hepatic phospholipid remodeling and modulates plasma triglyceride metabolism [46]. These findings elucidate the central regulatory role of the PPARδ-ACSL4/lysophosphatidylcholine acyltransferase 3 signaling axis in lipid metabolism and offer novel mechanistic insights into how PPARδ governs lipid metabolic networks by promoting arachidonoyl-CoA synthesis.

#### SREBP2

In HCC, SREBP2—a master transcription factor governing cholesterol biosynthesis—directly binds to the ACSL4 promoter region, inducing its transcriptional upregulation. Concurrently, the protein kinase B (Akt) signaling pathway facilitates ACSL4 packaging and secretion into extracellular vesicles via phosphorylation, revealing a dual “transcriptional activation–secretion enhancement” mechanism of the SREBP2/Akt axis within the tumor microenvironment [38]. Notably, SREBP2 deficiency not only impairs cholesterol synthesis but also downregulates sterol regulatory element-binding protein-1c and related lipogenic genes [47], suggesting SREBP2 may indirectly modulate ACSL4 function through global lipid metabolic reprogramming, thus reinforcing its central role in lipid homeostasis.

Additional transcriptional regulation: Besides PPARδ and SREBP2, other transcriptional regulators contribute to ACSL4 control. In liver cancer, ACSL4 regulation involves the cAMP and p38 mitogen-activated protein kinase (MAPK) pathways [48]. Zinc finger E-box binding homeobox 2, an epithelial-mesenchymal transition-inducing transcription factor, directly activates ACSL4 expression by binding to its promoter, establishing a positive feedback loop [49].

### Post-transcriptional regulation

#### microRNA

Multiple miRNAs directly or indirectly regulate ACSL4 expression, influencing tumor progression, ferroptosis, and metabolic processes. For instance, in triple-negative breast cancer, epigenetically silenced miR-449 family members enhance chemotherapy sensitivity by downregulating ACSL4, exhibiting the therapeutic potential of miRNA-mediated ACSL4 suppression [40]. In HCC, the ZNF8–miR–552-5p axis suppresses ACSL4 expression by targeting its 3′UTR, thus reducing ferroptosis susceptibility [50]. In addition, miR-424-5p and miR-4291 directly inhibit ferroptosis by targeting ACSL4 [41, 51].

Beyond direct regulation, miRNAs may indirectly modulate ACSL4 through PPAR signaling pathways. For example, miR-148a and miR-17-5p regulate milk fat synthesis by targeting PPARA and PPARGC1A [52], respectively, suggesting that PPAR family members mediate indirect miRNA regulation of ACSL4.

#### circRNA

CircRNAs function as competitive endogenous RNAs to post-transcriptionally regulate ACSL4 by sequestering miRNAs or binding RNA-binding proteins, thus modulating ferroptosis, tumor progression, and metabolic disorders in various diseases [53, 54]. In cervical cancer, circLMO1 acts as a molecular sponge for miR-4291 to relieve its suppression on ACSL4, thus promoting ferroptosis and inhibiting tumor growth [41]. Similarly, downregulation of circSCN8A in non-small cell lung cancer enhances miR-1290-mediated inhibition of ACSL4, inducing ferroptosis and suppressing tumor proliferation and metastasis [55]. Furthermore, in sepsis-associated acute lung injury, circEXOC5 stabilizes ACSL4 mRNA by interacting with polypyrimidine tract-binding protein 1, thus exacerbating ferroptosis [56].

Although studies on direct circRNA regulation of ACSL4 in liver diseases are limited, circRNAs play significant roles in non-proliferative chronic liver conditions (e.g., ALD, MASLD, viral hepatitis, liver injury/regeneration, cirrhosis, and autoimmune liver diseases) [54]. In HCC, circRNAs influence tumor proliferation, migration, and cell death resistance through ceRNA mechanisms or interactions with RNA-binding proteins [57]. Given the pivotal roles of ACSL4 in lipid metabolism and ferroptosis, further exploration of circRNA-mediated regulatory mechanisms may reveal novel therapeutic targets for liver diseases and cancer.

#### M^6^A modification

M^6^A is the most abundant internal chemical modification in eukaryotic mRNAs; it dynamically regulates gene expression by influencing multiple stages of mRNA metabolism, such as splicing, nuclear export, translation, and stability. This modification is catalyzed by methyltransferase complexes and can be reversibly removed by demethylases, reflecting its dynamically reversible nature. Recent studies have shown that m^6^A modification plays critical roles in various biological processes, such as cell differentiation, immune response, and tumorigenesis. Its dysregulation is closely associated with multiple diseases, making it a central focus in epitranscriptomics research [58-61]. In HCC, multiomics analyses have revealed significant associations of m^6^A modification with ferroptosis-related gene expression, implicating its regulatory role in ferroptosis susceptibility [62]. A mechanistic study revealed that lncRNA CBSLR, through the m^6^A–YTH domain family protein 2 (YTHDF2) axis, enhances the binding affinity of YTHDF2 to m^6^A sites on CBS mRNA. This action promotes the degradation of CBS mRNA, leading to a reduction in CBS protein levels. This decrease subsequently diminishes ACSL4 protein methylation, triggering polyubiquitination-mediated degradation and ultimately inhibiting ferroptosis in gastric cancer cells [63]. Original studies have further delineated the direct modification of ACSL4 mRNA by m^6^A “writer” enzymes across the following pathological contexts. In sepsis-associated acute lung injury, methyltransferase-like 3-mediated m^6^A modification enriches ACSL4 mRNA and enhances its stability via YTH domain-containing 1-dependent pathways, thus promoting ferroptosis and tissue damage [42]. In thoracic aortic aneurysms, methyltransferase-like 14 stabilizes ACSL4 transcripts by depositing m^6^A marks, which intensifies ferroptotic responses in vascular smooth muscle cells and underscores the critical role of m^6^A in vascular pathology [64]. Although current evidence regarding direct m^6^A modification of ACSL4 mRNA in liver injury remains limited, the multifaceted regulatory importance of m^6^A in gene expression underscores the potential research value of this field.

### PTMs

PTMs are essential enzymatic regulatory mechanisms that precisely modulate protein structure, stability, and functional activity through covalent modifications. In addition, they serve as central regulators in diverse biological processes and programmed cell death pathways, including ferroptosis [65, 66].

#### Ubiquitination

Ubiquitination is a crucial protein PTM that regulates substrate degradation or functional modulation through covalent ubiquitin conjugation [67, 68]. This modification plays a central regulatory role in the ACSL protein family, as exemplified by multiple mechanistic studies. Coactivator-associated arginine methyltransferase 1 (CARM1)-mediated methylation at R339 facilitates the interaction between the E3 ubiquitin ligase ring finger protein 25 and ACSL4, thus enhancing ACSL4 ubiquitination and subsequent degradation [69, 70].Conversely, eukaryotic translation initiation factor 3 subunit F directly interacts with ACSL4 to stabilize its protein levels via K48-linked deubiquitination, promoting lipid biosynthesis and tumor progression [44]. The lncRNA CBSLR promotes ACSL4 ubiquitination and degradation to inhibit ferroptosis [71], and ACSL4 stabilizes zinc finger E-box binding homeobox 2 by inhibiting its ubiquitin-mediated degradation, consequently promoting breast cancer progression [49]. These findings collectively demonstrate that ACSL4 protein levels and functions are precisely regulated by a dynamic ubiquitination–deubiquitination equilibrium. Notably, the deubiquitinase ubiquitin-specific peptidase 29 stabilizes ACSL5 by removing K48-linked ubiquitin chains, thus ameliorating MASLD progression [72], further highlighting the broad significance of ubiquitin-based regulation across the ACSL protein family.

#### Phosphorylation

Phosphorylation is another common PTM that alters protein conformation and function by adding phosphate groups to specific amino acid residues [65, 67]. ACSL4 was the first identified hormone-dependent phosphoprotein, and its dimerization enables phosphorylation by protein kinase A and protein kinase C, directly regulating its enzymatic activity [43]. In HCC, Akt-mediated phosphorylation of ACSL4 promotes its interaction with annexin A2 and enhances its packaging into large extracellular vesicles, thus influencing lipid reprogramming in neighboring cells [38]. Conversely, the phosphatase phospholysine phosphohistidine inorganic pyrophosphate phosphatase suppresses Akt activity, reducing ACSL4 phosphorylation at T624 and increasing cellular sensitivity to ferroptosis [73], which highlights the critical role of dephosphorylation in ACSL4 regulation.

Furthermore, ACSL4 regulation may involve the p38 MAPK pathway, a classic kinase cascade whose core mechanism relies on phosphorylation modifications [48, 74]. These studies show that the phosphorylation status of ACSL4 not only affects its enzymatic activity but also modulates protein–protein interactions, subcellular localization, and secretion processes, playing a pivotal role in lipid metabolism, tumor progression, and ferroptosis.

**Figure 1:**
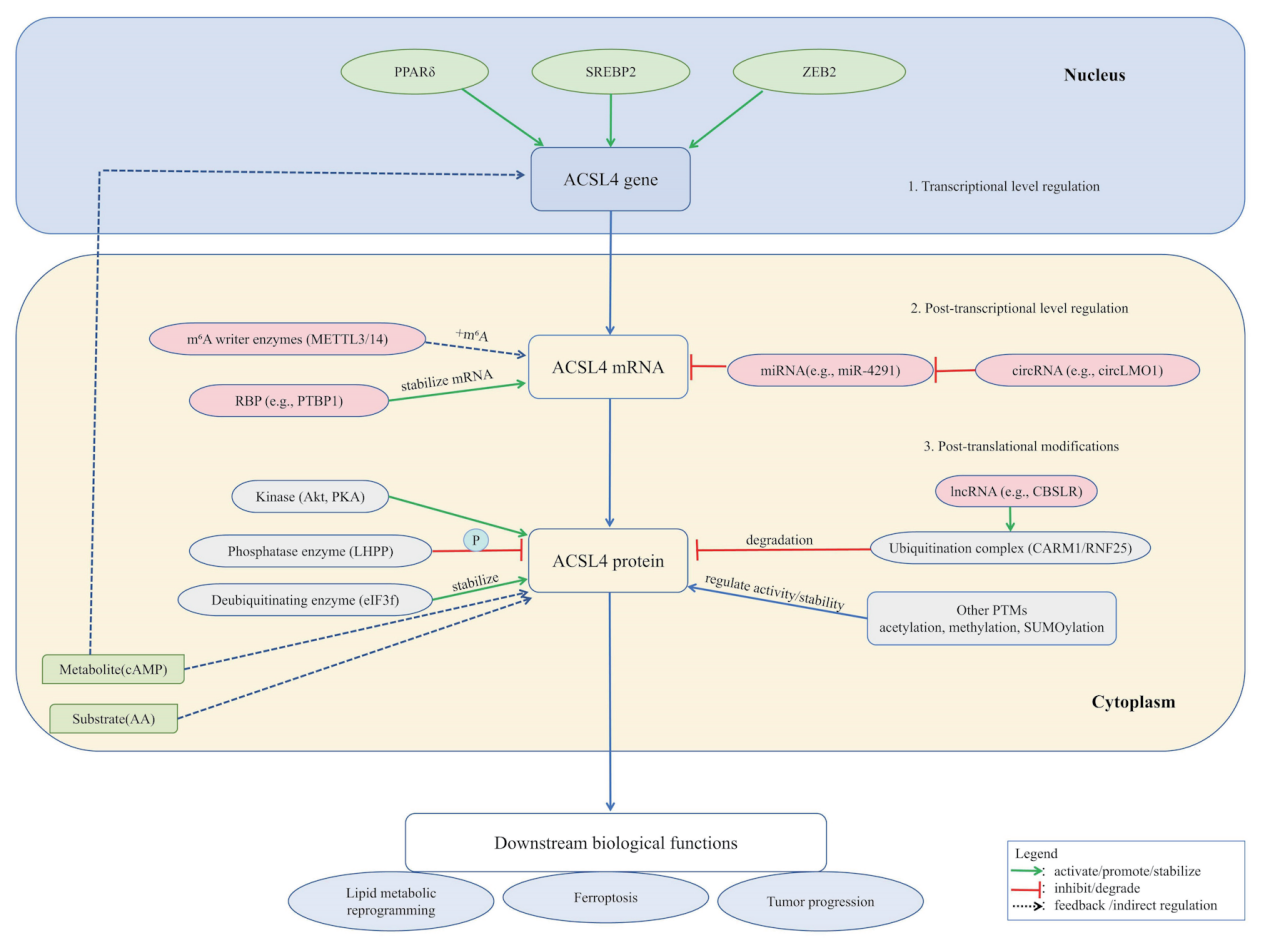
ACSL4 expression and function are subject to sophisticated, multi-layered regulation. At the transcriptional level, factors including PPARδ, SREBP2, and ZEB2 directly govern its gene expression. Post-transcriptionally, mRNA stability is enhanced by m^6^A modification (catalyzed by METTL3/14 complex) and RNA-binding proteins such as PTBP1, while non-coding RNAs (e.g., miR-4291 and circLMO1) exert regulatory effects through the ceRNA mechanism. At the post-translational level, ACSL4 protein activity is modulated by various modifications: ubiquitination (mediated by the CARM1/RNF25 complex and lncRNAs, such as CBSLR) and deubiquitination (e.g., eIF3f) regulate its stability; phosphorylation (e.g., Akt/PKA and LHPP) controls its function; additionally, modifications such as acetylation, methylation, and SUMOylation further fine-tune its activity and interactions. These integrated regulatory mechanisms collectively determine ACSL4’s biological roles in lipid metabolic reprogramming, ferroptosis, and tumor progression. ACSL4: acyl-CoA synthetase long-chain family member 4; PPARδ: peroxisome proliferator-activated receptor delta; SREBP2: sterol regulatory element-binding protein 2; ZEB2: zinc finger E-box binding homeobox 2; m^6^A: N6-methyladenosine; METTL3/14:methyltransferase-like 3/14; PTBP1: polypyrimidine tract-binding protein 1; Akt: protein kinase B; PKA: protein kinase A; LHPP: phospholysine phosphohistidine inorganic pyrophosphate phosphatase; eIF3f: eukaryotic translation initiation factor 3 subunit F; AA: arachidonic acid; cAMP: cyclic adenosine monophosphate; CARM1: coactivator-associated arginine methyltransferase 1; RNF25: ring finger protein 25.

Besides ubiquitination and phosphorylation, ACSL4 undergoes various PTMs. For instance, histone acetyltransferase 1/sirtuin 3-mediated acetylation at K383 enhances its protein stability by inhibiting f-box protein 10-mediated K48-linked ubiquitination [75]. ACSL4 can be modified by O-GlcNAc transferase on serine/threonine residues, with O-GlcNAcylation increasing its protein levels and promoting HCC cell proliferation and anti-apoptotic activity [76]. The coactivator CARM1 mediates R339 methylation of ACSL4. This modification promotes the binding of ACSL4 to the E3 ubiquitin ligase ring finger protein 25 and induces ubiquitination, which inhibits ferroptosis; therefore, blocking CARM1 enhances ferroptosis sensitivity [70]. TRIM28-mediated SUMOylation of ACSL4 regulates ferroptosis in neurons and cooperates with ubiquitination and autophagy pathways [77]. ACSL4 can reportedly undergo lysine propionylation under high-glucose or diabetic wound conditions, which promotes ferroptosis and autophagy in keratinocytes and accelerates wound healing-related pathways [78]. KAT3B (p300/CBP)-mediated succinylation at K661 enhances ACSL4 protein stability and modulates ferroptosis during cerebral ischemia–reperfusion injury [79]. These modifications precisely regulate the interactions, protein stability, and functions of ACSL4, participating in ferroptosis and various biological processes through sophisticated regulatory mechanisms (Fig. 1).

## Expression Profile and Localization of ACSL4 in Hepatic Cell Types

ACSL4 is expressed across multiple hepatic cell types, including hepatocytes, hepatic stellate cells (HSCs), Kupffer cells (KCs), and liver sinusoidal endothelial cells. Its subcellular localization is primarily observed in the mitochondrial matrix, endoplasmic reticulum membrane, and peroxisomal membrane. The cell type-specific expression levels and functional specialization of ACSL4 collectively form a sophisticated regulatory network that governs its diverse roles in both physiological and pathological processes in the liver.

### Expression and function in liver parenchymal cells

Hepatocytes, the primary functional cells of the liver, perform essential physiological functions, such as substance metabolism, biotransformation, and bile secretion. ACSL4 is constitutively expressed in hepatocytes, with both its expression levels and enzymatic activity being precisely regulated by nutritional status, hormonal levels, and pathological factors.

Under physiological conditions, ACSL4 participates in hepatic lipid metabolism by specifically catalyzing the esterification of long-chain PUFAs, such as AA and AdA, which incorporate them into membrane phospholipids and triglycerides [17, 80]. This process is essential for maintaining cell membrane fluidity, signal transduction, and energy storage.

Under pathological conditions like MASLD and its progressive form MASH, ACSL4 expression is often significantly upregulated [81]. Upregulated ACSL4 reportedly promotes the incorporation of PUFAs (particularly AA) into phosphatidylethanolamine, thus increasing the susceptibility of cellular membranes to lipid peroxidation and serving as a key driver of ferroptosis [81-83]. In liver tissues from MASLD/MASH patients and high-fat diet-induced animal models, elevated ACSL4 expression in hepatocytes was found to be strongly correlated with increased levels of ferroptosis markers and the degree of liver injury [37]. For example, one study revealed that hepatocyte-specific ACSL4 knockout significantly alleviated hepatic steatosis, inflammation, and ferroptosis in methionine–choline-deficient diet-induced MASH mouse models [84]. Furthermore, ACSL4 may exacerbate oxidative stress damage in hepatocytes by impairing mitochondrial function and promoting reactive oxygen species (ROS) production [85]. Emerging evidence suggests that ACSL4-mediated lipid reprogramming not only drives ferroptosis but also engages in crosstalk with other cell death pathways (e.g., apoptosis, necroptosis) to collectively exacerbate liver injury [33]. Notably, ACSL4 upregulation is reportedly also associated with HCC progression, potentially through mechanisms involving extracellular vesicle-mediated ACSL4 transfer to adjacent hepatocytes to induce senescence, or via p21-activated kinase 2 gene activation to promote HCC development [38, 86]. Consequently, pathological overexpression and activation of ACSL4 in hepatocytes is a critical pathophysiological mechanism underlying various liver injuries (including MASLD/MASH, DILI, and LIRI), making ACSL4 inhibition a promising therapeutic strategy for MASLD/MASH treatment [84, 87].

### Expression in HSCs and fibrosis-related functions

HSCs are the primary extracellular matrix-producing cells in the liver [88]. Under normal physiological conditions, they remain in a quiescent state and store vitamin A. Upon persistent liver injury, HSCs become activated and transform into myofibroblast-like cells, which excessively synthesize and secrete extracellular matrix components, such as collagen, leading to the initiation and progression of liver fibrosis [88, 89]. ACSL4 expression is relatively low in quiescent HSCs but significantly upregulated in activated HSCs [90]. Pharmacological or genetic enhancement of the ACSL4-mediated ferroptosis pathway can reportedly selectively eliminate activated HSCs, thus alleviating liver fibrosis. The underlying mechanisms involve the following pathways. Baicalin inhibits HSC activation through the miR-3595/ACSL4 axis to exert anti-fibrotic effects [91]; YTHDF2 regulates ACSL4 expression in an m^6^A-dependent manner to influence HSC ferroptosis and liver fibrosis progression [92]; finally, ginsenoside Rg3 reduces ACSL4 methylation through the miR-6945-3p/DNA methyltransferase 3 beta pathway to promote HSC ferroptosis and attenuate liver fibrosis [93]. However, the specific role of ACSL4 in HSCs and its net effect on liver fibrosis may depend on the disease stage, microenvironment complexity, and interactions with other signaling pathways. Targeted modulation of ACSL4 activity or ACSL4-mediated ferroptosis in HSCs provides novel therapeutic strategies for liver fibrosis treatment.

### Expression in KCs and inflammatory regulation

KCs, the liver-resident macrophages, are the first line of defense in the hepatic immune system. They play pivotal roles in pathogen recognition, clearance of necrotic cell debris, and inflammation regulation [94, 95]. KCs show remarkable heterogeneity and are distinguishable by their origins (e.g., embryo-derived resident KCs vs. monocyte-derived KCs) and activation states (e.g., classical M1 pro-inflammatory phenotype vs. alternatively activated M2 anti-inflammatory/repair phenotype) [95, 96].

ACSL4, a key enzyme regulating lipid metabolic reprogramming, specifically catalyzes the CoA esterification of PUFAs like AA. These activated PUFAs are subsequently incorporated into membrane phospholipids, and they provide essential substrates for downstream cyclooxygenase/lipoxygenase pathways to generate pro-inflammatory eicosanoids like prostaglandin e_2_ and leukotriene b_4_ [33, 97]. In myeloid-specific Acsl4 knockout mice, resident macrophages showed reduced synthesis of AA-derived lipid mediators and downregulated mRNA expression of pro-inflammatory factors such as Il6 and Nos2 [97]. Thus, evidence shows that ACSL4 promotes KC polarization toward M1-like pro-inflammatory phenotypes through lipid metabolic reprogramming, thus exacerbating hepatic inflammatory injury. In addition, ACSL4 may regulate KC functions via ferroptosis pathways. In KCs, ACSL4-mediated ferroptosis can trigger the release of pro-inflammatory damage-associated molecular patterns (e.g., high mobility group box 1), which exacerbate liver inflammation and injury in malaria models with hemozoin accumulation [98, 99]. Similarly, in systemic sclerosis models, ACSL4-induced KC ferroptosis promotes fibrotic progression [100]. However, the role of ferroptosis is highly context-dependent as scavenger receptor B class I-enriched exosomes alleviate excessive inflammation by inducing M1 macrophage ferroptosis in tumor microenvironments [101].

**Figure 2:**
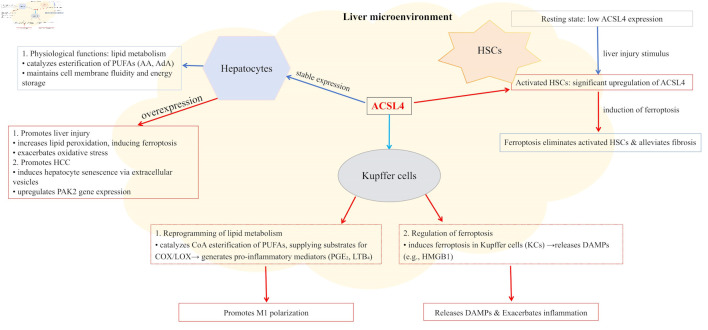
ACSL4 displays distinct expression levels and functions across different liver cell types, localizing to mitochondria, endoplasmic reticulum, and peroxisomes. In hepatocytes, upregulated ACSL4 expression promotes lipid peroxidation, thereby inducing ferroptosis, exacerbating oxidative stress, and driving HCC development. In Kupffer cells, ACSL4 catalyzes the esterification of PUFAs to provide substrates for the synthesis of pro-inflammatory mediators (e.g., PGE2 and LTB4) to promote M1 polarization, while also inducing ferroptosis and the release of DAMPs such as HMGB1. In HSCs, ACSL4 is significantly upregulated during activation, and inducing ferroptosis to clear activated HSCs can alleviate liver fibrosis. Within the holistic liver microenvironment, ACSL4 coordinately regulates lipid metabolism, inflammatory responses, and cell fate decisions. ACSL4: acyl-CoA synthetase long-chain family member 4; AA: arachidonic acid; AdA: adrenic acid; DAMPs: damage-associated molecular patterns; HCC: hepatocellular carcinoma; HSCs: hepatic stellate cells; MASLD: metabolic dysfunction-associated steatotic liver disease; PUFAs: polyunsaturated fatty acids; PGE2: prostaglandin E2; LTB4: leukotriene B4; HMGB1: high-mobility group box 1.

These findings suggest that ACSL4 jointly influences KC inflammatory phenotypes through two pathways, namely lipid metabolic reprogramming and ferroptosis regulation. Selective targeting of these pathways according to specific disease contexts may emerge as a novel therapeutic strategy for liver inflammation-related disorders (Fig. 2).

## Specific Roles of ACSL4 in Various Types of Liver Injury

### MASLD/MASH

ACSL4 plays multifaceted regulatory roles in the pathological progression of MASLD/MASH, wherein its aberrant expression and activity drive disease progression by promoting hepatic lipid accumulation, lipotoxic injury, inflammatory responses, and fibrosis. ACSL4 expression is reportedly significantly elevated in the livers of MASLD/MASH patients, and it activates ferroptosis through the c-Myc–ACSL4 pathway to accelerate disease deterioration [102]. The ACSL4-mediated activation of PUFAs is a key molecular mechanism—its catalyzed acyl-CoA products are incorporated into membrane phospholipids, becoming substrates for lipid peroxidation that directly trigger ferroptosis [28]. Regarding hepatic lipid accumulation, the selective ACSL4 inhibitor abemaciclib reduces lipid accumulation by enhancing mitochondrial respiration and fatty acid β-oxidation [37]. Conversely, ACSL4 knockout alleviates MASLD progression by downregulating the p38 MAPK/ GPX4 pathway to reduce lipid peroxidation [103]. In terms of lipotoxicity, upregulated ACSL4 in arsenic exposure and other MASH models promotes 5-hydroxyeicosatetraenoic acid (5-HETE) synthesis to exacerbate lipotoxic injury, whereas ACSL4 inhibition significantly mitigates this process [104]. Notably, the effects of ACSL4 on inflammation and fibrosis exhibit context-dependent characteristics. For instance, some studies found that hepatocyte-specific ACSL4 deletion showed no significant impact on MASLD progression in choline-deficient high-fat diet or Western diet models [105], whereas in perfluorooctane sulfonate-induced MASH models, ACSL4 markedly promoted liver injury and inflammatory responses by activating ferroptosis pathways [106]. These findings not only elucidate the central role of ACSL4 in MASH but also provide theoretical foundations for developing novel therapeutic approaches.

### ALD

Ethanol metabolism generates ROS through the cytochromeP450 2E1–microsomal ethanol oxidizing system and upregulates transferrin receptor 1/ferritinophagy, leading to hepatic iron overload that synergistically drives ferroptosis [107-110]. As a key executor of ferroptosis, the activity and expression of ACSL4 are positively regulated by lipid peroxidation products, forming a vicious cycle. Studies show significantly elevated ACSL4 protein levels in the Gao-binge alcohol model [111], whereas the ferroptosis inhibitor ferrostatin-1 or selenium supplementation alleviates liver injury by downregulating ACSL4 and upregulating GPX4/solute carrier family 7 member 11 [112]. Notably, ferrostatin-1 exerts superior protective effects compared to other inhibitors *in vitro*, further confirming the central role of ferroptosis in ALD [111]. Mechanistically, alcohol-induced ROS accumulation and PUFA peroxidation depend on the catalytic activity of ACSL4, with its knockout or inhibition significantly reducing lipid peroxidation and cell death [113]. In addition, caveolin-1 modulates ferroptosis through the ACSL4/GPX4/solute carrier family 7 member 11 pathway, an effect reversible by the ferroptosis agonist erastin [114]. These findings establish ACSL4 as a novel therapeutic target for ALD and provide new clinical intervention strategies.

### DILI

Multiple forms of DILI can exert their effects through the ACSL4-mediated ferroptosis pathway. In an acetaminophen (APAP) overdose-induced mouse model of acute liver failure, it was reported that n-6 polyunsaturated fatty acid radical oxidation drives ferroptosis through ACSL4-mediated lipid peroxidation, representing a key mechanism of APAP hepatotoxicity [115]. Activation of the nuclear factor erythroid-2-related factor 2 signaling can suppress ferroptosis and thus alleviate APAP-induced liver injury, a mechanism that may partially depend on the downregulation of ACSL4 expression and inhibition of lipid peroxidation [116]. Furthermore, studies report that anti-tuberculosis drugs significantly elevate ACSL4 levels both *in vivo* and *in vitro*, accompanied by Fe^2+^ accumulation and increased lipid peroxidation; conversely, the ferroptosis inhibitor ferrostatin-1 effectively reverses this process and mitigates liver injury [117]. These findings collectively indicate that ACSL4 is a critical mediator of hepatotoxicity induced by various drugs, and targeting this pathway holds promise as a novel strategy for the prevention and treatment of DILI.

### LIRI

In LIRI, ferroptosis is significantly activated, characterized by increased malondialdehyde, Fe^2+^, ROS, and glutathione depletion [118]. In fatty liver I/R models, ACSL4 and 4-hydroxynonenal (4-HNE) levels are markedly elevated, whereas the ferroptosis inhibitor liproxstatin-1 can mitigate injury by downregulating ACSL4 [119]. Furthermore, glycoprotein 78 deficiency significantly alleviates I/R injury by suppressing ACSL4-dependent ferroptosis [120], further confirming ACSL4 as a key regulatory target in I/R-associated ferroptosis.

### Viral hepatitis and liver fibrosis/cirrhosis

During the progression of viral hepatitis (hepatitis A virus (HAV)/HBV/HCV), ACSL4-mediated ferroptosis contributes to hepatic injury. This process occurs through mitochondrial dysfunction and lipid peroxidation, with its mechanisms closely linked to iron metabolism disorders (e.g., hepcidin/ferritin dysregulation) [118]. In the dynamic evolution of liver fibrosis, ACSL4 shows dual regulatory characteristics. The m^6^A reader protein YTHDF2 promotes ACSL4 translation by recognizing m^6^A modifications on ACSL4 mRNA, increasing ACSL4 protein expression, and inducing HSC ferroptosis to exacerbate fibrotic progression [92]. Conversely, ginsenoside Rg3 reduces ACSL4 methylation via miR-6945-3p-mediated DNA methyltransferase 3 beta inhibition, restoring ACSL4 expression and inducing HSC ferroptosis to alleviate fibrosis [93]. Traditional Chinese medicine interventions (e.g., Taohong Siwu Decoction) can mitigate fibrosis by regulating ACSL4-dependent lipid metabolism and mitophagy [121], suggesting that targeting ACSL4-regulated ferroptosis is a novel anti-fibrotic strategy, which allows for simultaneously eliminating activated HSCs while blocking chronic inflammation–fibrosis transition by improving iron overload and oxidative stress microenvironments.

### HCC

In HCC, ACSL4 shows complex “double-edged sword” characteristics, with its mechanisms of action closely tied to the tumor microenvironment. Studies show that ACSL4 reprograms lipid metabolism through the c-Myc/SREBP1 pathway, promoting triglyceride accumulation and tumor proliferation/invasion, which significantly correlates with poor patient prognosis [122]. However, ACSL4 is also a key mediator of sorafenib-induced ferroptosis; sorafenib triggers ferroptosis in HCC cells via ACSL4-dependent lipid peroxidation and glutathione depletion, with ACSL4 expression levels predicting drug sensitivity [123, 124]. Artemether synergistically enhances this effect when combined with sorafenib [125].

Regarding drug resistance mechanisms, the ZNF8–miR-552-5p axis suppresses ACSL4 expression by targeting its 3′UTR and thus reducing HCC cell susceptibility to ferroptosis [50]. Similarly, the ferroptosis inducer erastin increases lipid peroxidation products (e.g., malondialdehyde) and inhibits proliferation by downregulating ACSL4 expression [126]. Pancreatic cancer models also show that ADP-ribosylation factor 6 knockdown upregulates ACSL4, which significantly enhances RAS-selective lethal 3-induced lipid peroxidation and cell death while reversing gemcitabine resistance [127]. Furthermore, ACSL4 regulates intercellular interactions within the HCC microenvironment. HCC-derived exosomal miR-142-3p induces M1 macrophage ferroptosis by targeting solute carrier family 3 member 2. This promotes tumor proliferation, migration, and invasion, effects that are reversible by miR-142-3p knockdown or solute carrier family 3 member 2 overexpression [128].

These findings suggest that precision therapeutic strategies targeting ACSL4 require context-specific design: either inhibiting its pro-tumor functions or selectively activating its pro-ferroptotic effects, offering novel approaches for HCC treatment.

## ACSL4-Targeted Interventions and Therapeutic Strategies

ACSL4, a key enzyme in the execution of ferroptosis, has emerged as an attractive therapeutic target because of its central role in various liver injuries. Current intervention strategies primarily focus on the development of small-molecule inhibitors, genetic-level regulation, and combination therapies.

### Small-molecule inhibitors and drug screening

Duan et al first reported that low-dose abemaciclib could specifically inhibit ACSL4 activity, which reduced lipid peroxidation and fat accumulation and thus ameliorated liver inflammation and fibrosis in MASLD mouse models [37]. Similarly, thiazolidinedione drugs like rosiglitazone suppress ACSL4 in a PPARγ-independent manner, regulate fatty acid metabolism, and mitigate arsenic-induced MASH and ferroptosis by lowering 5-HETE levels [129, 130]. In addition, the small-molecule candidate AS reportedly directly binds ACSL4 at Gln464 to inhibit its catalytic function, with its nanoformulation showing broad-spectrum anti-ferroptosis effects in both renal ischemia–reperfusion and acute liver injury models [131]. Conversely, while the pan-ACSL inhibitor triacsin C effectively inhibits triacylglycerol synthesis in hepatocytes and macrophages, it also triggers mitochondrial stress and apoptosis; this highlights the safety and efficacy advantages of ACSL4-specific inhibitors [132]. These findings provide multi-layered theoretical foundations and candidate molecules for developing novel ACSL4-targeted therapies.

### Genetic knockout/knockdown and siRNA strategies

Recent studies have shown that ACSL4-targeted genetic knockout or RNA interference techniques exert multiple biological effects through distinct mechanisms. In liver disease models, ACSL4 deficiency significantly attenuates hepatic fibrosis and cell proliferation by suppressing ferroptosis, thus delaying HCC progression [133]. In arsenic-induced MASH models, ACSL4 siRNA inhibits ferroptosis by reducing 5-HETE levels [104]. Metabolic studies reveal that adipocyte-specific ACSL4 knockout ameliorates obesity-related metabolic disorders and enhances energy metabolism by decreasing AA incorporation into phospholipids and 4-HNE generation [87]. Conversely, liver-specific ACSL4 deletion reduces VLDL-TG secretion but causes lysophospholipid accumulation and insulin resistance [46]. Notably, ACSL4 deficiency shows no significant metabolic improvements in choline-deficient high-fat diet or Western diet-induced MASLD models, suggesting that its effects may be regulated by dietary and microbial factors [105]. These findings suggest that the regulatory roles of ACSL4 have marked tissue specificity and pathological context-dependence, providing crucial theoretical foundations for developing targeted therapeutic strategies.

### Combination therapies: synergistic strategies with antioxidants and autophagy modulators

Studies show that in diabetic liver injury, palmitic acid induces hepatic damage through ACSL4-dependent ferroptosis and autophagy inhibition. Notably, autophagy modulators (3-MA/rapamycin) regulate ferroptosis sensitivity by mediating lysosomal degradation of ACSL4 [134]. The threonine-protein kinase 1 inhibitors KW-2449 and necrostatin-1 both suppress ferroptosis by inhibiting UNC-51-like kinase 1-dependent autophagy [135]. Further research reveals that ferritinophagy modulation effectively reduces ACSL4-mediated lipid peroxidation damage [136]. In the treatment of hepatic and intestinal ischemia–reperfusion injury, the lipid antioxidant liproxstatin-1 significantly lowers alanine aminotransferase (ALT)/aspartate aminotransferase (AST) levels and lipid peroxidation products (e.g., 4-HNE); it achieves this by inhibiting the ACSL4-dependent ferroptosis pathway [119, 137]. These findings support an innovative multi-target combination strategy: synergistically targeting ACSL4 (specific inhibitors), modulating the ferritinophagy pathway, and applying lipid antioxidants. This comprehensive approach can regulate iron metabolism, lipid peroxidation, and autophagy. Ultimately, this combined strategy offers novel therapeutic insights and intervention targets for various liver diseases, including diabetic hepatopathy and fatty liver-associated injury.

### Clinical research and transformation strategy

To our knowledge, although no direct ACSL4 inhibitors have yet entered clinical trials for liver diseases, drugs that indirectly modulate its expression or ferroptosis processes (e.g., thiazolidinediones) have shown therapeutic efficacy in specific hepatic disorders [129, 130]. Current challenges include developing highly specific and low-toxicity ACSL4 inhibitors/modulators, achieving liver-targeted delivery to reduce systemic toxicity, and establishing personalized treatments based on a patient’s ACSL4 expression and iron metabolism status. Concurrently, in-depth elucidation of the role and regulatory networks of ACSL4 across different liver injury stages will inform precision intervention strategies. Regarding translational opportunities, high ACSL4 expression in HCC patients shows significant correlation with sorafenib treatment response rates (66.7% vs. 23.5%), suggesting its potential as a therapeutic sensitivity biomarker [124]. Multicenter cohort studies further confirm that ACSL4 overexpression is an independent risk factor for poor HCC prognosis (hazard ratio (HR) = 4.23, 95% confidence interval (CI), 1.19–15.04) and is significantly associated with post-TACE recurrence risk, highlighting its clinical value for prognostic stratification [138, 139].

Against this backdrop, the specific ACSL4 inhibitor PRGL493 shows potential therapeutic value across multiple disease contexts. By inhibiting ACSL4 activity and blocking the conversion of AA to AA-CoA, PRGL493 has shown efficacy in diverse disease models, including suppression of tumor growth, reduction of steroid hormone synthesis, reversal of tumor drug resistance, and attenuation of pathological progression in conditions such as breast cancer [140, 141], pulmonary fibrosis [142], endometriosis [143], severe acute pancreatitis [144], and HCC [145]. In liver-specific studies, PRGL493 has been shown to mitigate lipid peroxidation and oxidative damage induced by tumor necrosis factor-α and palmitic acid following extensive hepatectomy, thereby alleviating liver injury and improving survival rates [146]. These findings further support the feasibility of incorporating ACSL4 inhibitors into the aforementioned multi-target therapeutic strategy. However, current research on PRGL493 remains limited to the preclinical stage, and its clinical translation requires further exploration to validate the safety and efficacy of this multi-target combination strategy.

## Conclusions

ACSL4 has emerged as a key lipid-metabolizing enzyme with preferential catalytic activity toward AA and AdA. In recent years, its multidimensional regulatory networks, pathophysiological functions, and therapeutic potential in liver injury and related hepatic diseases have become a research hotspot, indicating significant clinical translation prospects. The expression and activity of ACSL4 are precisely regulated at multiple levels, including transcriptional, post-transcriptional, and PTMs. This regulation involves various transcription factors, non-coding RNAs, and mechanisms like ubiquitination and phosphorylation, among others. Dysregulation of these regulatory networks is closely associated with the pathogenesis and progression of liver diseases.

ACSL4 shows cell-type-specific expression patterns and functions in different hepatic cells. In hepatocytes, ACSL4 plays a dual role in regulating lipid metabolism and ferroptosis. Under physiological conditions, it maintains cell membrane homeostasis, whereas in pathological states, it promotes lipid peroxidation and ferroptosis [46, 147, 148]. In HSCs, ACSL4 expression increases as the cells become activated. This process is driven by YTHDF2-mediated m^6^A modification, which stabilizes the *ACSL4* mRNA and promotes fibrogenesis [92, 149]. ACSL4 regulates KC functions through a dual mechanism. This involves catalyzing PUFA esterification to drive M1 polarization via the production of pro-inflammatory mediators while simultaneously modulating inflammatory responses through ferroptosis induction; the net effect shows significant context dependence [97, 100, 101].

Although ACSL4 participates in hepatic lipid metabolism remodeling through its canonical enzymatic activity, under specific pathological conditions, it also profoundly influences hepatocyte fate and disease progression, particularly by catalyzing PUFA esterification to drive ferroptosis [8, 150]. In various liver disease models, including MASLD/MASH [81, 151], ALD [152], DILI [153, 154], and LIRI [155], ACSL4-mediated ferroptosis is widely recognized as a key mechanism underlying hepatocyte damage and death. During the complex progression of liver fibrosis and HCC, ACSL4 shows dual roles. Although it may promote disease progression by activating HSCs or stimulating HCC cell proliferation [91, 133], targeted induction of ACSL4-dependent ferroptosis in activated stellate cells is a potential antifibrotic strategy [93, 156]. Similarly, inducing ACSL4-dependent ferroptosis in cancer cells may suppress tumor growth [157].

Currently, small-molecule inhibitors targeting ACSL4 (e.g., triacsin C, liproxstatin-1, and abemaciclib) and RNA interference technologies have shown promising results *in vitro* and in animal models [104, 119, 132]. However, related clinical research remains in its early stages. Future research directions should focus on 1) developing novel ACSL4 inhibitors with higher selectivity and improved oral bioavailability; 2) optimizing drug delivery systems like nanocarriers or liposomes to achieve liver-specific or cell-specific targeted delivery; 3) actively exploring biomarkers based on ACSL4 expression levels or functional states for clinical prognosis evaluation and treatment efficacy monitoring; and finally, 4) further investigating the synergistic therapeutic potential of combining ACSL4 inhibitors with antioxidants, autophagy modulators, or drugs targeting other key signaling pathways. This approach may yield more effective multifaceted intervention strategies for liver injury and related hepatic diseases.

Taken together, as a critical regulator of hepatic lipid metabolism and ferroptosis, ACSL4 plays a central role in the pathophysiology of various liver injuries and related diseases. A deeper understanding of its complex regulatory mechanisms and multifaceted functions will provide new theoretical foundations, potential therapeutic targets, and innovative treatment strategies for the diagnosis and management of liver diseases.

## Data Availability

The authors declare that data supporting the findings of this study are available within the article.

## Abbreviations

4-HNE: 4-hydroxynonenal
5-HETE: 5-hydroxyeicosatetraenoic acid
AA: arachidonic acid
ACSL4: acyl-CoA synthetase long-chain family member 4
AdA: adrenic acid
Akt: protein kinase B
ALD: alcoholic liver disease
APAP: acetaminophen
cAMP: cyclic adenosine monophosphate
CARM1: coactivator-associated arginine methyltransferase 1
CoA: coenzyme A
DILI: drug-induced liver injury
GPX4: glutathione peroxidase 4
HAV: hepatitis A virus
HBV: hepatitis B virus
HCC: hepatocellular carcinoma
HCV: hepatitis C virus
HSCs: hepatic stellate cells
KAT3B: lysine acetyltransferase 3B
KCs: Kupffer cells
LIRI: liver ischemia-reperfusion injury
m^6^A: N6-methyladenosine
MAPK: mitogen-activated protein kinase
MASH: metabolic dysfunction-associated steatohepatitis
MASLD: metabolic dysfunction-associated steatotic liver disease
MAM: mitochondria-associated endoplasmic reticulum membrane
PPAR: peroxisome proliferator-activated receptor
PPARA: peroxisome proliferator-activated receptor alpha
PPARδ: peroxisome proliferator-activated receptor delta
PPARγ: peroxisome proliferator-activated receptor gamma
PPARGC1A: peroxisome proliferator-activated receptor gamma coactivator 1 alpha
PTMs: post-translational modifications
PUFAs: polyunsaturated fatty acids
ROS: reactive oxygen species
SREBP2: sterol regulatory element-binding protein 2
YTHDF2: m^6^A–YTH domain family protein 2
